# Does insurance protect individuals from catastrophic payments for surgical care? An analysis of Ghana’s National Health Insurance Scheme at Korle-Bu teaching Hospital

**DOI:** 10.1186/s12913-020-4887-2

**Published:** 2020-01-17

**Authors:** Juliet Okoroh, Doris Ottie-Boakye Sarpong, Samuel Essoun, Robert Riviello, Hobart Harris, Joel S. Weissman

**Affiliations:** 1 Brigham and Women’s Hospital, Center for Surgery and Public Health, Boston, MA USA; 2 Department of Surgery, Korle-Bu Teaching Hospital, Accra, Ghana; 3 National Institute of Health, Fogarty International Center- GLOCAL Consortium, Bethesda, USA; 4 Department of Surgery, University of Alabama, 1720 2nd Ave S KB 217, Birmingham, AL 35294-0016 USA; 5 University of Ghana, Accra, Ghana; 6 Dodowa Health Research Centre, Accra, Ghana; 7 Department of Surgery, University of California San Francisco, San Francisco, CA USA

**Keywords:** Catastrophic health expenditures, Surgical care, Universal health coverage, Ghana

## Abstract

**Background:**

According to the World Health Organization, essential surgery should be recognized as an essential component of universal health coverage. In Ghana, insurance is associated with a reduction in maternal mortality and improved access to essential medications, but whether it eliminates financial barriers to surgery is unknown. This study tested the hypothesis that insurance protects surgical patients against financial catastrophe.

**Methods:**

We interviewed patients admitted to the general surgery wards of Korle-Bu Teaching Hospital (KBTH) between February 1, 2017 – October 1, 2017 to obtain demographic data, income, occupation, household expenditures, and insurance status. Surgical diagnoses and procedures, procedural fees, and anesthesia fees incurred were collected through chart review. The data were collected on a Qualtrics platform and analyzed in STATA version 14.1. Fisher exact and Student T-tests were used to compare the insured and uninsured groups. Threshold for financial catastrophe was defined as health costs that exceeded 10% of household expenditures, 40% of non-food expenditures, or 20% of the individual’s income.

**Results:**

Among 196 enrolled patients, insured patients were slightly older [mean 49 years vs 40 years *P* < 0.05] and more of them were female [65% vs 41% *p* < 0.05]. Laparotomy (22.2%) was the most common surgical procedure for both groups. Depending on the definition, 58–87% of insured patients would face financial catastrophe, versus 83–98% of uninsured patients (all comparisons by definition were significant, *p* < .05).

**Conclusion:**

This study—the first to evaluate the impact of insurance on financial risk protection for surgical patients in Ghana—found that although insured patients were less likely than uninsured to face financial catastrophe as a result of their surgery, more than half of insured surgical patients treated at KBTH were not protected from financial catastrophe under the Ghana’s national health insurance scheme due to out-of-pocket payments. Government-specific strategies to increase the proportion of cost covered and to enroll the uninsured is crucial to achieving universal health coverage inclusive of surgical care.

**Trial registration:**

Registered at www.clinical trials.gov identifier NCT03604458.

## Background

### Essential and emergency surgery in universal health coverage

It is estimated that 11–33% of the global burden of disease can be treated surgically; and this includes conditions related to cancer, obstetrics, congenital anomalies, and injury [1–4]. Yet, access to affordable healthcare remains a significant challenge globally. An estimated 3.7 billion individuals are at risk for catastrophic health expenditures (CHEs) due to surgical care, with a disproportionate burden in Sub-Saharan Africa and South-East Asia [5]. In May 2015, the World Health Assembly passed resolution 68.15, which called for the recognition of emergency and essential surgery as integral components of achieving universal health coverage (UHC) [6]. The goal was to improve access, financial risk protection, as well as the responsiveness of health systems in averting disability and productivity loss due to surgical conditions. Pivotal to these efforts has been the growing emphasis of the role of insurance in strengthening health systems. Prepayment schemes such as health insurance effectively pool risk and can protect households against unforeseen health shocks [7]. In fact, health insurance has been associated with a reduction in out-of-pocket expenditures (OOPEs) in Low-Middle Income Countries (LMICs) like Indonesia, Mali, China, India, Columbia, and Rwanda [8, 9]. With ongoing recommendations by the Lancet Commission on Global Surgery to develop national surgical plans in LMICs that provide 100% coverage against CHE(s) by 2030, it is important to understand how health insurance schemes are designed to improve access to surgical care, particularly in countries undergoing health care reform [10].

### The experience of Ghana with universal health coverage

Ghana is a low middle income country with a population of 27 million people whose national health insurance scheme (NHIS) was implemented in 2003 [11]. NHIS is a government-sponsored mandatory insurance plan which is funded by the National Health Insurance Levy, a value added tax on all goods and services in the country [11]. This comprises 75% of its funding; the rest is from premiums, social security deductions, and registration fees [11–13]. NHIS covers 95% of conditions affecting Ghanaians and includes a variety of inpatient and outpatient services in surgery, obstetrics, general medicine, and emergency care [14]. Premiums are assessed based on ability to pay and typically range from 7.2 Ghana Cedis (US$2.88) to 48 Cedis (US$19.18) [15]. Premium exemptions exist for specific groups such as individuals above 70 years of age, the physically or mentally disabled, pregnant women, children [15], Social Security and National Insurance Trust SSNIT pensioners, indigents, and recipients of Government of Ghana cash grant [16]. This comprises close to 60% of the insured population who are exempt from paying premiums and registration fees. As of 2017, 35% of the Ghanaian population was actively enrolled in the scheme with ongoing plans by the government to expand enrollment of the uninsured [17]. NHIS has been praised for its efforts in achieving equity and inclusion in health insurance schemes in LMICs and has served as a model for many countries in Sub-Saharan Africa that are undergoing health reform [18].

### Impact of insurance on health costs and outcomes in Ghana

Analysis of the NHIS of Ghana over the past 10 years has demonstrated a positive impact on maternal health, with mother’s more likely to receive antenatal care, to deliver at an institution, and to have assistance from a skilled birth attendant [19–25]. In addition, insured members are more likely to visit clinics, seek formal care when ill, and are more likely to obtain prescriptions [14]. With respect to the costs of healthcare, the probability of a household incurring CHEs was 4.2 times less likely for the insured than for the uninsured, although, insurance did not eliminate all direct costs associated with treatment [26]. In a prior systematic review, we found studies reporting up to 18% of insured households in Ghana would face financial catastrophe due to health costs [27]. In addition, gaps do exist in the coverage of medicines and supplies [27]. Although these studies provide the foundation for understanding the impact of NHIS on cost and utilization, there has been no study to date on the impact of NHIS on financial risk protection and cost of surgical care. Given the current emphasis of the role of surgery in UHC, our objective in this study was to test the hypothesis that insurance makes a difference in out-of-pocket expenditures for surgical care and CHE(s) by comparing rates of financial catastrophe for insured versus. Uninsured surgical patients at a single institution, the Korle-Bu Teaching Hospital (KBTH).

## Methods

### Study site/population

KBTH, founded in 1923, is the largest tertiary teaching hospital and the major referring hospital in Ghana. The hospital is situated in the southwestern part of Accra, the capital of Ghana. The hospital treats patients referred from centers all over Ghana and neighboring West African countries. Inhabitants are mostly urban or suburban with a small proportion of rural and slum dwellers. The Department of Surgery at KBTH has four general surgical units and additional wards encompassing orthopedic surgery, plastic surgery, urology, and neurosurgery. In 2016, a total of 7941 operations were performed; 33% in trauma, 30% in general surgery, 12% pediatric surgery, 8% neurosurgery, 6% urology, and 5% in ophthalmology/oral maxillary facial surgery. The top three general surgery diagnoses in 2016 were symptomatic hernia, appendicitis, and breast cancer [28].

### Study design

We conducted a cross-sectional survey of a random sample of patients who were admitted and discharged on the general surgery ward between February 1st 2017 and October 1st 2017 in the Department of General Surgery at KBTH.

### Sampling method

Efforts were made to sample across all four general surgical wards to obtain a representative sample of inpatients. Data collection consisted of two essential components: First, prior to discharge, patients on the general surgery wards were interviewed by two trained research assistants using a questionnaire developed based on the 2014 Ghana Demographic Health Survey and administered in the respondent’s language [29]. It included modules on the respondent’s demographic profile (age, ethnicity, income, employment status, occupation), socio-economic status, household characteristics (number of people in the household, sex of head of household, number of children, household expenditures on food, non-food expenditures), dwelling characteristics, as well as assets ownership. The health utilization module included number of hospitalizations during the last 3 months, number of out-patient visits, and the presence of chronic medical conditions [29]. Participants were also interviewed to collect payment information and receipts for items not billed by the surgical department but incurred as part of the hospitalization. This included payments for laboratory tests, imaging, and medicines billed by other departments or an outside facility. From this, we calculated the total cost to the patient for the hospitalization. Non-medical costs (indirect costs) were calculated as the cost of transportation to the facility and estimated lost wages, both obtained via the respondent’s interview. The lost wages were derived from the average daily wages, obtained from the respondent’s interview, multiplied by the length of stay for the hospitalization, obtained from the hospital record. The questionnaire is available in the appendix. The second component was a chart review and abstraction by the research assistants to obtain all costs of inpatient surgical care. From the hospital record, the primary surgical and any medical diagnoses were obtained as well as all procedures performed. Procedural fees, anesthesia fees, consultation fees, supplies, accommodations fees, and all hospital costs incurred were collected. The surgical cost minus NHIS payments was considered the OOPE for the insured at the surgery department level. The same analysis was repeated for charges outside the surgery department. The uninsured paid 100% of all costs as out-of-pocket payments. The OOPEs are defined from the patient’s perspective, i.e. the cost to the patient or the amount paid by the patient to the provider i.e. the hospital for all related health expenses that is not reimbursed by NHIS.

### Sample size

For the primary comparison of financial catastrophe by insurance status, we assumed the insured would be less likely to make catastrophic payments. Previous studies on non-surgical respondents indicated that 6% of the insured compared to 28% of the uninsured made catastrophic payments [27]. Using this difference and a sample ratio of insured to uninsured respondents of 3 to 1, 80% power, and a 95% confidence interval at the level of 0.05 yielded a sample size of 98 participants (25 uninsured, 73 insured) to detect a significant difference in financial catastrophe by insurance status. This sample size was calculated using the OpenEpi, Version 3 (2008) open source calculator-Proper [30].We sampled 203 respondents in order to have power for further subgroup analysis and to account for the possibility of non-response. A total of 196 patients had complete variables of interest (96% response rate) and were included in the final analysis.

### Data description and analysis

We used Stata 14.0 to generate frequencies, means and proportion utilizing the Fisher Exact tests. Multiple logistic regression models were used to obtain the relationships between financial catastrophe, and socio-demographic and clinical characteristics. An alpha level of 0.05 was used to determine the statistical significance. We analyzed all available observations and records. Observations and records with missing information on specific variables of interest were excluded from the analysis. Lastly, the principal component analysis was used to generate a household wealth index using 22 household living items including dwelling characteristics, access to utilities, and ownership of household items.

Statistical analysis was conducted at the individual and household levels. At the individual level, we examined the impact of NHIS on OOPEs. The OOPEs for the insured were calculated as the sum of: 1) total direct surgery department cost minus calculated payments to be made by NHIS and 2) payments made to other departments and facilities. For the uninsured, NHIS made no contributions and this was the total direct medical cost. Indirect costs were obtained from the transportation cost and calculated lost wages (individual’s reported daily wage multiplied by the length of hospital stay). We compared the mean OOPEs between the insured and uninsured using descriptive statistics. We also compared the means of the socioeconomic and clinical characteristics between the insured and uninsured using Student t tests. Fisher Exact tests were used to identify and compare the frequencies of all variables between the insured and uninsured.

At the household level, we explored the impact of NHIS on financial catastrophe using accepted definitions in the literature [31–35]. OOPEs were considered catastrophic if they exceeded 10% of annual total household expenditures, 20% of the individual's income, or 40% of non-food expenditures. Multiple regression models were developed to identify associations between financial catastrophe and socio-demographic and clinical characteristics. The questionnaire was administered and data collected through Qualtrics platform software by research assistants using a secured tablet. Data from the review of the hospital records and payment receipts for all costs incurred were also collected in Qualtrics platform software. The data were managed in Microsoft Excel 2014 and analyzed using Stata 14.1. An alpha level of 0.05 was used to determine the statistical significance.

### Permission and ethical considerations

We sought ethical approval from both the University of California San Francisco and Korle-Bu Teaching Hospital. Permission was further obtained from the Department of the General Surgery at KBTH. Informed consent was also obtained from all participants in the study in order the ensure confidentiality by not disclosing any personal identifiers or names in data capturing, analysis and report writing.

## Results

### Background characteristics

There were 127 (65%) respondents in the insured group and 69 (35%) in the uninsured group. In terms of demographic and household characteristics, only age, sex, marital status, and number of children differed significantly between the two groups (Tables 1 and 2). Of note, 36% of the respondents were unemployed; they reported ill health, being retired, and educational activities as reasons for not working. The average monthly income was GH¢ 754.0 (167 USD) and this did not differ by insurance status (Table 3). Average monthly expenditures were GH¢ 841 (186 USD), and non-food expenditures were GH¢ 620 (138 USD). On average, the insured and uninsured were similar in terms of community factors, such as distance to the nearest healthcare facility, although the insured made, on average, three visits to a health facility and the uninsured made two visits in the 3 months preceding the survey (*p* = 0.003) (Table 4). The most common operation performed was a laparotomy (22%) due to 1) intestinal obstruction, 2) peritonitis from perforated peptic ulcer/viscus, and 3) obstructive jaundice. The next most common operations were appendectomy (14%) and inguinal hernia repair (10%). Other procedures performed such as extremity or soft tissue surgery accounted for 13.0% of all procedures performed. Thirteen percent of respondents did not require

**Table 1:** Background characteristics of study respondents

Variables	Total
Individual Characteristics % (N)	
Age (years)	
Mean age	47.2 (SD± 1.188)
<25	9.2% (18)
25-39	24.5% (48)
40-69	54.6% (107)
≥ 70	11.7% (23)
Sex	
Male	43.9% (86)
Female	56.1% (110)
Ethnicity	
Akan	45.9% (90)
Ga-Dangme	23.5% (46)
Ewe	15.3% (30)
Other	15.3% (30)
Marital Status	
Never married	20.4% (40)
Currently married	54.1% (106)
Formerly married	25.5% (50)
Education	
None	11.2% (22)
Primary	26.0.% (51)
Secondary	42.9% (84)
Tertiary	19.9% (39)
Employment Status	
Unemployed	36.2% (71)
Employed	63.8% (125)
Occupation	
Unemployed	36.2% (71)
Formal	11.2% (22)
Informal	52.5% (103)
Had a Procedure	
Yes	83.2% (163)
No	13.3% (26)
Missing	7.5% (7)
Household Characteristics (mean)	
Household size	6.2 (SD±0.348)
Number of children	2.8 (SD±0.140)
Sex of the house-hold head %(N)	
Male	66.8% (131)
Female	18.4% (36)
Missing	14.8% (29)

**Table 2:** Bivariate analysis of health insurance status of respondents by background characteristics

Variables	Health insurance status (*N* = 196)		*P*-value (Fisher exact test)
	Insured 65% (*N* = 127)	Uninsured 35% (*N*=69)	
Age (years)			
<25	6.3%(8)	14.5% (10)	**0.034**
25-39	20.4% (26)	31.9% (22)	
40-69	59.1% (75)	46.4% (32)	
≥ 70	14.2% (18)	7.2% (5)	
Sex			
Male	35.4% (45)	59.4%( 41)	**0.002**
Female	65.6% (82)	41.6% (28)	
Ethnicity			
Akan	41.7% (53)	53.6% (37)	0.496
Ewe	16.6% (21)	13.1% (9)	
Ga-Dangme	25.2% (46)	20.3 %(14)	
Other	16.5% (30)	13.0% (9)	
Marital Status			
Never married	15.0% (19)	30.4% (21)	0.037
Currently married	57.5% (73)	47.8% (33)	
Formerly married	27.5% (35)	21.8% (15)	
Education			
None	11.8% (15)	10.1% (7)	0.434
Primary	28.3% (36)	21.8% (15)	
Secondary	38.6% (49)	50.7% (35)	
Tertiary	21.3% (27)	17.4% (12)	
Employment Status			
Unemployed	38.6% (49)	31.9% (22)	0.437
Employed	61.4% (78)	68.1% (47)	
Occupation			
Unemployed	38.6% (49)	31.9% (22)	0.608
Formal	10.2% (13)	13.0% (9)	
Informal	51.2% (65)	55.1% (38)	
Had a Procedure			
Yes	87.8% (101)	85.3% (52)	0.644
No	12.2% (14)	14.7% (9)	
Household Characteristics (mean)			
House-hold size	6.5	5.6	0.21
Number of children	3.1	2.2	**0.003**
Sex of household head			
Male	76%(84)	82.5 (47)	0.43
Female	23%(26)	18 (10)

**Table 3:** Welfare Characteristics of the Respondents

Variables	Health insurance status		*P*- value (Fisher’s exact test)	95% CI
	Insured	Uninsured		
Individual Gh¢ ($USD)				
Mean monthly income	744.5 (78)	800.64 (47)	0.529	680.5 - 850.7
House-hold Gh¢ ($USD)				
Mean monthly total expenditures	904.3 (127)	726.9 (69)	0.027	766.3-917.4
Mean monthly non-food expenditures	668.4. (127)	531.4 (69)	0.033	559.6-680.8
Household wealth index %(N)^a^				
Poor	33% (43)	38% (26)	0.471	
Middle	30%(38)	35% (24)		
Rich	36%(46)	28% (19)		

Source: Authors; computed from individual survey data, 2017

Ghana Cedis (Gh¢) 4.5= $1.0USD, March 2018

T- test for equal means, significant level at 0.05

^a^Based on principal component analysis of the response of individuals to 22 items which included households’ dwelling characteristics, access to utilities and ownership of consumer durables. (N) number of respondents

**Table 4:** Health Systems and Community Factors by Insurance Status

Variable	Health insurance status		*P*-value (Fischer’s exact test)
	Insured	Uninsured	
Regular health care provider % (N)			
Yes	86.5 (109)	76.1 (51)	0.074
No	13.5 (17)	23.9 (16)	
Type of health facility			
Tertiary/Teaching Hospital	45.7 (58)	42.0 (29)	0.871
Public/Quasi/Private Hospital	19.7 (25)	23.2 (16)	
Polyclinic	28.3 (36)	30.4 (21)	
Other	6.3 (8)	4.4 (3)	
Distance to home facility			
<1 KM-10 KM	11.8 (15)	13.0 (9)	0.526
11-15KM	16.6 (21)	17.4 (12)	
16-20KM	41.7 (53)	49.3 (34)	
>20 KM	29.9 (38)	20.3 (14)	
Healthcare utilization			
Mean number of health facility visits (in last three months)	3.1	2.2	0.003

a surgical procedure. There was no significant difference in the number and types of operations performed by insurance status.

### Direct and indirect costs of surgical care

Table 5 presents the mean direct costs and indirect costs of surgical care by insurance status. The average departmental cost per patient for surgical care with a procedure was GH¢ 997 ($222 USD) and without a procedure was GH¢ 478.3 ($106 USD). Average cost of ancillary services, which included medicines, laboratory tests, and imaging, was GH¢ 1079 ($240 USD). The average total direct cost of surgical care (direct OOPE) was GH¢ 2819 (626 USD). The insured paid 1.74 times less than the uninsured [GH¢ 2239 ($497 USD) vs GH¢ 3887 ($863 USD) (*p* = 0.0001)]. The non-medical expenditures – which included costs such as transportation costs and indirect costs such as lost wages – were GH¢ 234 ($52.0 USD) on average and did not differ by insurance status (*p* = 0.657).

**Table 5:** Cost of Surgical Care and Financial Catastrophe by Insurance Status

	Health insurance status		*P*-value (Fischer’s exact test)
	Insured	Uninsured	
Out of pocket (OOP) expenditures Gh¢ ($USD)			
Mean OOP health expenditure (direct medical cost for surgery)	997.1 (203)	2,860.0	<0.001
Mean OOP health expenditures (direct medical cost for ancillary services)	1100 (244)	1,039.0	0.667
Mean OOP non-medical cost/indirect health costs (transportation and lost wages)	243 (54)	218.0	0.657
Total OOP health expenditures (direct & indirect costs)	2238.7 (497)	3,887.5 (863)	<0.001
Financial Catastrophe %^a,b^			
Percent total OOP out of individual income	40%	61%	0.037
Percent total OOP out of house-hold expenditures	25%	53%	<0.001
Percent total OOP out of subsistence expenditures^c^	67%	142%	<0.001
Percentage of individuals incurring catastrophic payments^d^			
Percentage of individuals incurring catastrophic health payments (income level)	63%	83%	0.017
Percentage of individuals incurring catastrophic health payments (subsistence level)	58%	94%	<0.001
Percentage of individuals incurring catastrophic health payments (house-hold level)	87%	98%	0.008

^a^Financial catastrophe is defined as out of pocket payment for surgery which exceeds; 20% of the respondent’s income, 10% of the household expenditures, or 40% of the household’s net-food expenditures

^b^Percent of the respondent’s annual income, house-hold expenditures, or subsistence expenditures that was spent on surgical care . i.e. the insured spent on average 40% of their annual income on surgical care

^c^Subsistence expenditures: Total annual household expenditures minus food expenditures

^d^Percentage of individuals incurring catastrophic health payments by insurance status at different catastrophic levels i.e. 63% of the insured incurred catastrophic payments for surgical care at the income level

### Catastrophic health expenditures by insurance status

Table 5 depicts financial catastrophe by insurance status using total OOPE, defined as payment in excess either of 20% of the individual's income, 10% of household expenditure, or 40% of non-food expenditures. On average, in our study population, the insured spent 39% of their estimated annual income on surgical care, whereas the uninsured spent 61% (*p* < 0.04). We also found significant differences in the percentage of individuals making catastrophic payments by insurance status measured at different catastrophic thresholds. Using the 20% of individual income definition, 63% of the insured compared to 83% of the uninsured made catastrophic payments (*p* < 0.02). Using the 10% of the household expenditure definition, 87% of the insured compared to 98% of the uninsured made catastrophic payments (*p* < 0.008). Finally, using the 40% of subsistence expenditures definition, 58% of the insured compared to 94% of the uninsured made catastrophic payments (*p* < 0.001).

### Determinants of catastrophic health expenditures

The multiple logistic regression estimates for all individuals demonstrated that significant determinants of CHEs are insurance status, having a surgical procedure, education attainment, and household wealth index. The results also showed that participating in NHIS reduces the probability of incurring CHEs. At the income level, uninsured members were six times more likely to incur CHEs for surgical care (OR 5.52; 95% CI: 1.70–17.92) than the insured. For those living at the subsistence level, the uninsured were 16 times more likely to incur CHEs. Although factors such as age > 40, female sex, being married, or working in the informal sector were associated with an increased likelihood of CHEs, none were statistically significant at the 0.05 alpha level.

## Discussion

Our study—which examines the extent to which Ghana’s NHIS protects individuals needing surgical care from financial catastrophe—shows that surgery is still unaffordable for more than 60% of insured patients seen at KBTH. This is a significant problem because one in every three respondents lives within the lowest wealth quintile and risk impoverishment as a consequence of paying for surgery. The costs associated with receiving surgical care at KBTH, for the insured, constitute 33% of the GDP per capita income as of 2016 ($1507 USD) [36]. Likewise, OOPEs for the insured at KBTH constitute 60% of their total health expenditures, which greatly exceeds the WHO recommendations for OOPEs not to exceed more than 20% of total health expenditures [37, 38].

We found that insurance was not associated with a difference in the cost of ancillary services such as medicines, laboratory testing, and imaging that are included in the NHIS. The NHIS medicine list includes 517 medications that are part of the WHO list of essential medications, which are supposed to be covered free at the point of care [39, 40]. Furthermore, routine laboratory tests and diagnostics such as ultrasound are supposed to be covered [41]. The findings we observed could in part be because the KBTH pharmacy does not accept NHIS payments and often patients have to obtain medicines outside the hospital [ 42]. Potential explanations for this phenomenon reported in the literature include anecdotal evidence of patients being told that the NHIS medicines are out of stock, poor reimbursement to the pharmacy by NHIS, and selection bias towards patients paying out of pocket [43]. Medicines such as antibiotics and pain medications, which are on the NHIS medicines list, but often not comprehensively covered, are essential components of surgical care and access to these medication needs to be addressed.

NHIS reimbursed on average 40% of the total cost of surgical care, which is based on the G-DRG. Anesthesia fees including the consultation fees and the cost of the anesthetic drugs are not reimbursed at the hospital level. This is a significant barrier because the vast majority of cases performed at KBTH in general surgery require anesthesia support. The anesthetic drugs are obtained from the pharmacy by the anesthesiologist and billed to the patient at the end of the procedure. This represents a potential opportunity for intervention. For example, NHIS should cover the cost of anesthesia care, and costs might be saved by improving the procurement practices at KBTH.

Our study has several limitations which must be acknowledged. Close to 70% of respondents in our study were insured, which is almost twice the national average for Ghana and could represent a selection bias toward insurance, and thus limits extrapolation of our findings to the national level [44]. This is partly because the Greater Accra region is among the top three regions with the highest insurance penetration [44]. In addition, KBTH, the largest hospital in Ghana, has a diverse socio-economic background, which makes it a representative sample of *urban* Ghana; but our findings may not be generalizable to *rural* areas. Next, we did not collect data on how households coped with making payments and the contributions of relatives and non-government institutions in reducing the financial burden of care. This could be significant because based on our calculation, 60% of patients would likely not be able to afford surgery without other means of hardship financing, despite having health insurance. In fact, one out of three households in Africa and Southeast Asia pay for medical expenses by borrowing, liquidation of assets, or selling [45, 46]. This type of hardship financing also increases the probability that a household would be in further debt as a result of seeking healthcare. Moreso, we were unable to collect indirect non-medical cost data associated with care-givers as our study focused on sampling the experience of patients primarily. Finally, further studies are needed to compare costs of surgery at KBTH to costs at other facilities as there have been no study to date on the cost of surgical care at other facilities in Ghana.

Another limitation is that we only captured patients requiring inpatient stay/admission; hence, our findings and costs would not be applicable for patients undergoing same-day or ambulatory surgery. We also are unable to capture the cost of foregone care, or delays in diagnosis and treatment, as our study only examined CHE(s) on a sample of patients who obtained surgical intervention at the hospital. We did not capture any associated costs for follow-up or readmissions for complications, although outpatient visits are paid for at capitated rates by NHIS [47], both of these would further drive up OOPE for patients.

Lastly, there might be limitations inherent in the survey technique used as it is often difficult to ask individuals to recall their income accurately, which may in some circumstances be seasonal or largely informal in LMICs [48, 49]. To account for this, we used the standardized GDHS and included three different catastrophic thresholds for which our results show internal consistency. We recognize that our study was not conducted at the household level, as is often the case in the GDHS, Ghana Living Standard Survey, and World Health Survey, but our target population was at the facility level [29, 50, 51]. Uniquely, our study provides the most sensitive and accurate cost estimates because we reviewed the actual cost of care to the patient at the facility. Prior studies in Ghana have been conducted through household surveys, which are limited by estimates of payments for health-care made by respondents and subject to recall bias [26, 27]. Those survey studies, however, also validate our findings of insured patients still at financial risk and paying out-of-pocket for services that are included in the scheme.

Our study findings lead us to offer several policy recommendations that could improve the equity in UHC as it relates to surgical care at KBTH. First, the calculated amount reimbursed for each surgical DRG is too low; in our study, the insurance paid on average 40% of the total cost of care and did not include many services. This fee-for-service model based on the DRG in Ghana for inpatient services is a flat reimbursement scheme with no price caps on total expenditures. Consequently, the hospital can shift more costs not reimbursed by NHIS to the patient which can constitute up to 40% of a patient’s annual income. This means of financing is not only regressive but also inconsistent with the core mission of the NHIS to provide a health insurance scheme that “*adequately covers against the need to pay out of pocket at the point of service”* [ 52]. Most health insurance schemes of high-income countries with an overall lower incidence of CHE(s) offer either no fees, a co-payment (a fixed amount for each covered service), co-insurance (a fixed percentage of the total claim amount), or a deductible (a fixed amount that patients must pay for services before insurance benefits apply). This is dependent on the benefit design and type of services provided, i.e., inpatient, outpatient, pharmacy [53–55]. Co-insurance typically ranges from 10% of inpatient cost in Japan and South Korea, to up to 30% in countries within the Organization for Economic Co-operation and Development [54, 55]. Germany, Sweden, and Switzerland require a flat copayment of between (9–12 USD) per day for hospital stays [55]. The UK, Canada, and Norway require no co-payment for inpatient services utilized at accredited facilities [55]. Therefore, government strategies to: 1) improve the reimbursement rates for surgical care, and 2) institute cost-sharing for surgical conditions at KBTH that is equitable, would overall reduce out-of-pocket payments particularly for individuals who cannot afford to pay.

Secondly, efforts towards health equity should include efforts to better identify individuals who cannot afford to make payments. This could be improved through local identification systems with proxy means testing at the point of care. This is typically done at the enrollment phase in Ghana, as 60% of the insured population are exempt from paying the annual premiums. However, despite this, insured poor households are not exempt from making payments at the point of care. Examples from high income countries include Germany’s health system, which provides a price cap whereby out-of-pocket cost should not exceed 1–2% of the annual household income. England, France, Israel, Italy, Japan, Sweden, Switzerland, Norway, the Netherlands, and the United States provide exemptions from cost sharing for low income and specific populations [55–57]. Furthermore, enhancing efforts through the use of the existing social services in enrolling the uninsured at the point of care could offer a potential opportunity to expand the risk pool, thereby generating further revenue for the health insurance scheme.

## Conclusion

This study—the first to analyze the impact of that National Health Insurance Scheme of Ghana on the cost of surgical care—shows that despite its benefits, surgery is still largely unaffordable for the majority of surgical patients seen at Korle-Bu Teaching Hospital. Though insured patients have greater financial risk protected than the uninsured, they continue making out-of-pocket payments for services that are intended to be included in the health insurance scheme. Greater than 50% of insured patients would face financial catastrophe to be able to receive surgical care. Our study summarizes the experience of the use of NHIS at a teaching hospital in Ghana, providing some evidence to guide policy reform in similar countries. Government-specific strategies to address these gaps in coverage are key to sustaining the mission of NHIS to provide universal health coverage inclusive of surgical care.

## Data Availability

The data that support the findings of this study are available from the corresponding author but restrictions apply because these data were used under license for the current study and are not publicly available. Data are, however, available from the authors upon reasonable request and with permission from Korle-Bu Teaching Hospital Ethical Committee and Institutional Review Board.

## Abbreviations

CHE: Catastrophic health expenditure
G-DRG: Ghana’s Diagnosis Related Grouping of the International Classification of Diseases 10th revision
KBTH: Korle-bu Teaching Hospital
LMIC: Low-and middle-income countries
NHIA: National Health Insurance Authority
NHIS: National Health Insurance Scheme of Ghana
OECD: Organization for Economic Co-operation and Development
OOP: Out of pocket payment
OOPE: Out of pocket expenditures
UHC: Universal health coverage
WHA: World Health Assembly
WHO: World Health Organization
